# AlGaN/GaN Metal-Oxide-Semiconductor High-Electron-Mobility Transistor with Polarized P(VDF-TrFE) Ferroelectric Polymer Gating

**DOI:** 10.1038/srep14092

**Published:** 2015-09-14

**Authors:** Xinke Liu, Youming Lu, Wenjie Yu, Jing Wu, Jiazhu He, Dan Tang, Zhihong Liu, Pannirselvam Somasuntharam, Deliang Zhu, Wenjun Liu, Peijiang Cao, Sun Han, Shaojun Chen, Leng Seow Tan

**Affiliations:** 1 College of Materials Science and Engineering, Shenzhen Key Laboratory of Special Functional Materials, Nanshan District Key Lab for Biopolymer and Safety Evaluation, Shenzhen University, 3688 Nanhai Ave, Shenzhen, 518060, People Republic of China; 2 State Key Laboratory of Functional Materials for Informatics, Shanghai Institute of Microsystem and Information Technology, CAS, 865 Chang Ning Road, Shanghai, 200050, People Republic of China; 3 Department of Physics, National University of Singapore, 21 Lower Kent Ridge Road, 117576, Singapore

## Abstract

Effect of a polarized P(VDF-TrFE) ferroelectric polymer gating on AlGaN/GaN metal-oxide-semiconductor high-electron-mobility transistors (MOS-HEMTs) was investigated. The P(VDF-TrFE) gating in the source/drain access regions of AlGaN/GaN MOS-HEMTs was positively polarized (i.e., partially positively charged hydrogen were aligned to the AlGaN surface) by an applied electric field, resulting in a shift-down of the conduction band at the AlGaN/GaN interface. This increases the 2-dimensional electron gas (2-DEG) density in the source/drain access region of the AlGaN/GaN heterostructure, and thereby reduces the source/drain series resistance. Detailed material characterization of the P(VDF-TrFE) ferroelectric film was also carried out using the atomic force microscopy (AFM), X-ray Diffraction (XRD), and ferroelectric hysteresis loop measurement.

GaN has become a very promising candidate for high voltage and high power applications^1^,^2^,^3^,^4^, mainly due to its wide energy bandgap *E*_*g*_ (3.4 eV), large conduction band offset Δ*E*_*C*_ between Al_*x*_Ga_1-*x*_N and GaN (up to 1.78 eV for AlN), high two-dimensional electron gas (2-DEG) density of the order of ~1 × 10^13^ cm^−2^, and high electron saturation velocity (up to 1.5 × 10^7^ cm/s)^5^,^6^,^7^,^8^,^9^,^10^,^11^. Substantial progress has been made in GaN power devices with the demonstration of off-state breakdown voltages of up to several kV^12^,^13^,^14^,^15^,^16^,^17^,^18^,^19^,^20^,^21^,^22^,^23^,^24^. The highest off-state breakdown voltage of 10.4 kV was achieved in AlGaN/GaN high electron mobility transistors (HEMTs) on sapphire with an on-resistance *R*_*on*_ of 186 mΩ·cm^2^^25^. On the other hand, although GaN power devices, such as AlGaN/GaN HEMTs, have achieved a lower on-resistance than that of silicon devices for a given off-state breakdown voltage, they have yet to achieve an on-resistance value close to the theoretical limit of GaN. Reasons for this could be the poor contact resistance between the ohmic contact and the AlGaN/GaN layer, and relatively high sheet resistance in the source/drain access regions.

At another front, it has been reported that a ferroelectric film, such as poly[(vinylidenefluoride-co-trifluoroethylene)] [P(VDF-TrFE)] and Pb(Zr, Ti)O_3_ (PZT), with a large remnant polarization *P*_*r*_ can modulate the 2-DEG density of the AlGaN/GaN heterostructure, without degrading the carrier transport within AlGaN/GaN heterostructure^26^,^27^. As shown in Ref. 26 and 27, the carrier density of AlGaN/GaN heterostructure with P(VDF-TrFE) or PZT can be significantly modulated by changing the external electric field. However, the effect of integrating P(VDF-TrFE) or PZT gating into AlGaN/GaN HEMTs (at a device level) has not been investigated so far. As shown in Table 1, compared to other ferroelectric materials (PbTiO_3_, SrBiNb_2_O_9_, and BiFeO_3_), P(VDF-TrFE) has a large coercive field *E*_*C*_ of 1.2 MV/cm and a large remnant polarization *P*_*r*_ of 4.8 μC/cm^2^. Especially, P(VDF-TrFE) can be deposited at a room temperature using a cost-effective spin coating method, as compared to other ferroelectric materials, which require a sophisticated vacuum system with a high temperature process, such as molecular beam epitaxy (MBE), liquid delivery metal organic chemical vapor deposition (LDMOCVD) etc. The advantage of depositing the P(VDF-TrFE) film at room temperature by spin-coating is to avoid the interfacial diffusion and chemical reaction between P(VDF-TrFE) and the underlying AlGaN layer, which normally happens in a high temperature deposition process^28^,^29^,^30^. These make P(VDF-TrFE) as an attractive material which can be integrated into the AlGaN/GaN HEMTs for performance enhancement. The ferroelectricity of the P(VDF-TrFE) film originates from the molecular dipoles associated with partially positively charged hydrogen (H) and partially negatively charged fluorine (F). The all-trans conformation of chain molecules and their parallel packing cause the alignment of all molecular dipoles in one direction, inducing a large spontaneous polarization when an external field is applied. The large coercive field of the P(VDF-TrFE) film also requires a large depolarization field to flip the dipole direction and ensures dipole stability.

In this article, the P(VDF-TrFE) ferroelectric polymer gating was applied on the AlGaN/GaN metal-oxide-semiconductor high-electron-mobility transistors (MOS-HEMTs) for the first time for the reduction of source/drain series resistance. The P(VDF-TrFE) film was deposited over the AlGaN/GaN MOS-HEMTs, and the P(VDF-TrFE) film in the source/drain access regions was positively polarized (i.e., partially positively charged hydrogen was aligned to the AlGaN surface) by an applied external electric field. When the P(VDF-TrFE) film is polarized, the aligned positively charged H can shift down the conduction band of the AlGaN/GaN heterostructure and increase the 2-DEG density, similar to the effect of a GaN/AlN/GaN triple cap layer on the AlGaN/GaN heterostructure^31^,^32^. Compared to the device with unpolarized P(VDF-TrFE) film, the source/drain series resistance *R*_*S/D*_ for device with polarized P(VDF-TrFE) film was reduced by 16%.

## Results and Discussion

**Figure 1:**
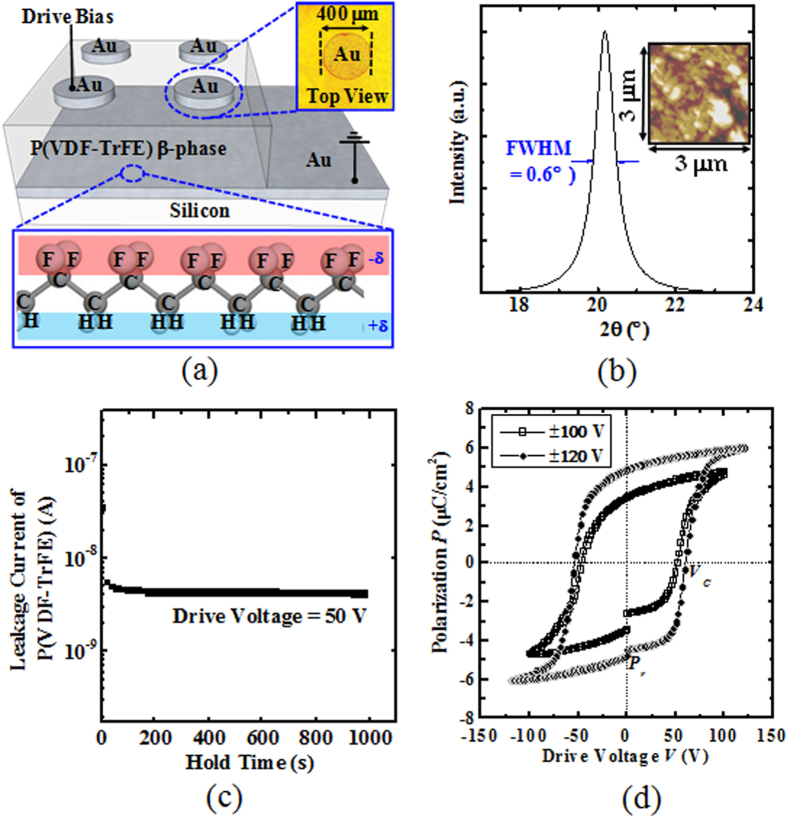
(**a**) Schematic of a P(VDF-TrFE) test structure: Au/P(VDF-TrFE)/Au/Si (The diameter *d* of the Au pad is 400 μm). Top-view of the test structure obtained by optical microscope is shown at the top-right corner. A schematic of the β-phase P(VDF-TrFE) is also shown at the bottom. (**b**) XRD and AFM (inset) scans of the P(VDF-TrFE) film after baking at 135 °C for 20 hours. A strong peak with a full width at half maximum (FWHM) of 0.6° located at ~20° indicates the formation of β-phase P(VDF-TrFE). The AFM scan shows the P(VDF-TrFE) film surface with a root-mean-square (RMS) roughness of 5 nm. (**c**) Leakage current of P(VDF-TrFE) measured using a Au/P(VDF-TrFE)/Au/Si test structure. The leakage current of P(VDF-TrFE) as a function of time was measured with a bias of 50 V applied between the top and bottom electrodes. (**d**) Polarization charge as a function of drive voltage (*P* – *V*) for the Au/P(VDF-TrFE)/Au/Si test structure. Remnant polarization *P*_*r*_ and coercive voltage *V*_*c*_ are 4.8 μC/cm^2^ and 60 V, respectively. The coercive field is *E*_*c*_ 1.2 MV/cm.

Circular test structures made of Au (80 nm)/P(VDF-TrFE) (500 nm)/Au (80 nm)/Si wafer were fabricated together with the AlGaN/GaN MOS-HEMTs. These test structures were used to measure the leakage current and the polarization of the P(VDF-TrFE) film. Figure 1(a) shows the schematics of these test structures and the β-phase P(VDF-TrFE), where partially negatively charged F and partially positively charged H of P(VDF-TrFE) are separately aligned on the opposite sides of the carbon molecular chain. The top view of the optical image of one of the test structure is also shown in Fig. 1(a). The P(VDF-TrFE) test structure was baked at 135 °C for 20 hours to form the crystalline β-phase P(VDF-TrFE), which was confirmed by a X-Ray Diffraction (XRD) scan as shown in Fig. 1(b). A strong peak with a full width at half maximum (FWHM) of 0.6° located at ~20° in the XRD scan indicates the formation of β-phase P(VDF-TrFE). In addition, the surface morphology of the P(VDF-TrFE) film was characterized using Atomic Force Microscopy (AFM). Based on the area size of 3 μm by 3 μm, the P(VDF-TrFE) film has a root-mean-square (RMS) roughness of 5 nm [inset of Fig. 1(b)]. The leakage current of the P(VDF-TrFE) test structure remained around 4.3 × 10^−9^ A when a voltage of 50 V was applied between the top and bottom electrodes Fig. 1(c). A low leakage current of the P(VDF-TrFE) film is essential for integration in the AlGaN/GaN MOS-HEMTs. Otherwise, surface leakage through the P(VDF-TrFE) film can degrade the device performance. Ferroelectric hysteresis loops of P(VDF-TrFE) was measured as a function of drive voltage as shown in Fig. 1(d). This was performed on the test structure by using a Radiant Technology Precision LC. From Fig. 1(d), remnant polarization *P*_*r*_ of 4.8 μC/cm^2^ (~charge density of 3.0 × 10^13^ cm^−2^) and coercive voltage *V*_*c*_ of 60 V were obtained. These values are close to the previously reported ones^33^,^34^.

**Figure 2:**
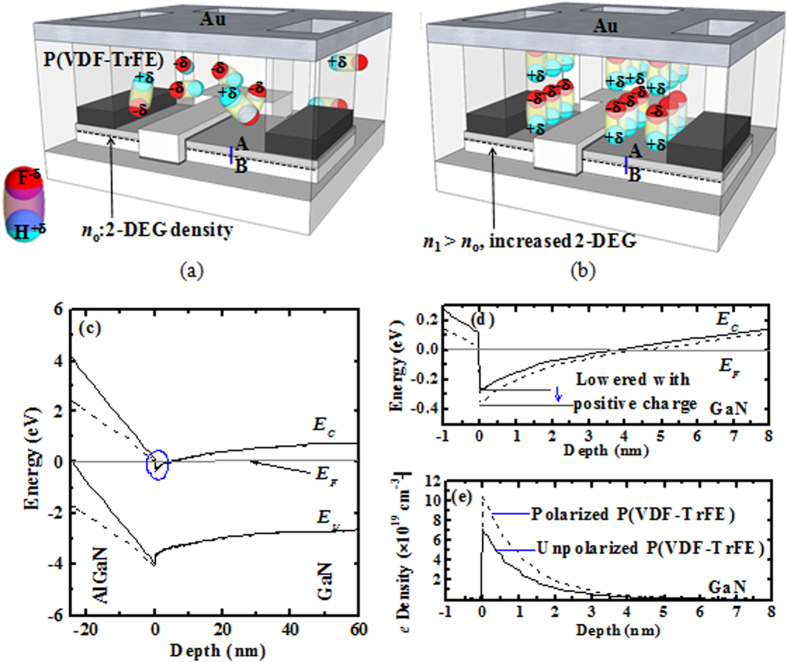
(**a**) Schematic of an AlGaN/GaN MOS-HEMT with an overlaying P(VDF-TrFE) film and a gold (Au) electrode. Without polarization, the dipoles in the P(VDF-TrFE) film are randomly distributed. The 2-DEG density of AlGaN/GaN heterostructure without polarization is *n*_0_. (**b**) Schematic of AlGaN/GaN MOS-HEMTs with a positively polarized P(VDF-TrFE) film (i.e. positively charged H atoms aligned to the AlGaN surface). The 2-DEG density with polarization for AlGaN/GaN heterostructure is *n*_1_, which is larger than the value of *n*_0_ as shown in (**a**). (**c**) Energy band diagram of the Al_0.25_Ga_0.75_N/GaN heterostructure from a TCAD simulation (Synopsys Sentaurus simulator) along the blue line AB [shown in Fig. 1(a,b)] with unpolarized (solid lines) and positively polarized (dash lines) P(VDF-TrFE) gating (Positive charge density 3.0 × 10^13^ cm^−2^ on the AlGaN surface is used in the calculation here). (**d**) Zoomed-in band alignment and (**e**) Electron distribution profile of the circled region in Fig. 1(c) in the access region of the AlGaN/GaN MOS-HEMT with unpolarized (solid lines) and polarized (dash lines) P(VDF-TrFE) gating.

**Figure 3:**
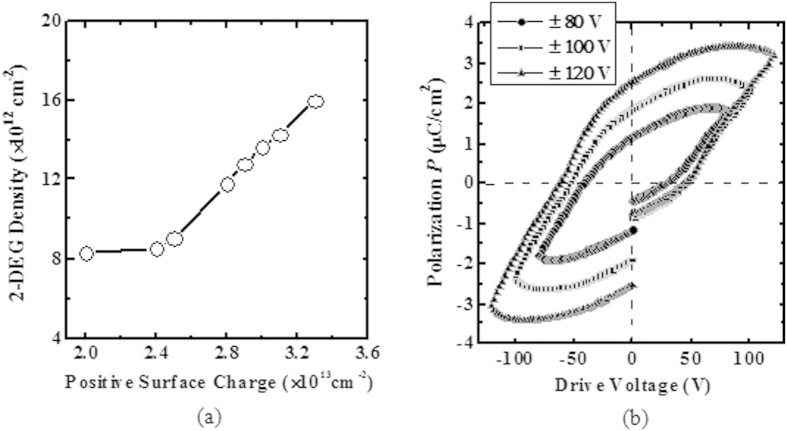
(**a**) Simulated 2-DEG density for Al_0.25_Ga_0.75_N(25 nm)/GaN heterostructure as a function of positive charge density on the AlGaN surface. (**b**) Polarization as a function of drive voltage (*P* – *V*), for AlGaN/GaN MOS-HEMTs with P(VDF-TrFE) gating. The voltage is biased between the Au electrode (grounded) and the source/drain pads. The Au electrode was grounded, and the drive voltage applied on the source/drain pads was swept from 0 V to the positive maximum voltage, then back to the negative maximum voltage, and then to 0 V.

As illustrated in Fig. 2(a), the P(VDF-TrFE) molecular dipoles are normally randomly distributed without polarization (i.e., partially positively charged H and partially negatively charged F are randomly aligned with respect to the AlGaN surface). Upon applying an external electric field across the P(VDF-TrFE) film (gold electrode is grounded and source/drain pads are negatively biased), partially positively charged H will be aligned to the AlGaN surface, resulting in a large spontaneous polarization which can increase the 2-DEG density of AlGaN/GaN heterostructure in the access regions [*n*_1_ >*n*_0_ as shown in Fig. 2(b)]. In order to understand the effect of the polarized P(VDF-TrFE) film on 2-DEG density, TCAD simulations were performed. The energy band diagram along the line AB [as shown in Fig. 2(a,b)] was examined for the AlGaN/GaN heterostructure with both polarized and unpolarized P(VDF-TrFE) films. Energy band alignments for the Al_0.25_Ga_0.75_N/GaN heterostructure along the blue line AB with unpolarized (solid lines) and polarized (dash lines) P(VDF-TrFE) film, calculated using the Synopsys Sentaurus simulator, is shown in Fig. 2(c). The polarized partially positively charged H of P(VDF-TrFE) film were treated as fixed positive charges with a density of 3.0 × 10^13^ cm^−2^ (~remnant polarization *P*_*r*_ of 4.8 μC/cm^2^) on the AlGaN surface in Fig. 2(c). Upon the application of an external electric field, the conduction band of AlGaN layer was bent downward due to the polarization of the P(VDF-TrFE) film. The zoomed-in view of the circled region in Fig. 2(c) is shown in Fig. Figure 2(d). The conduction band in both AlGaN and GaN regions is lowered, thereby increasing the 2-DEG density in the triangular quantum well at the AlGaN/GaN interface^35^. In addition, the electron distribution profiles for both cases are shown in Fig. 2(e). The electron density was enhanced after polarizing the P(VDF-TrFE) film. The 2-DEG density was obtained by integrating the electron density along the depth from AlGaN/GaN interface as shown in Fig. 2(e). In Fig. 3(a), the 2-DEG density was plotted as a function of the positive charge density on the AlGaN surface, and 2-DEG density is about 13.6 × 10^13^ cm^−2^ for the positive charge density of 3.0 × 10^13^ cm^−2^. With a larger amount of the polarized positive charge in P(VDF-TrFE) film over the AlGaN/GaN access regions, the 2-DEG density was further increased.

In following section, the electrical results of the AlGaN/GaN MOS-HEMTs with unpolarized and polarized P(VDF-TrFE) gating will be discussed. First of all, the P(VDF-TrFE) film was polarized by an applied external electric field. This was achieved by grounding the Au electrode, and applying a drive voltage on the source/drain pads. The drive voltage was swept first from 0 V to the positive maximum voltage, then back to negative maximum voltage, and then to 0 V, so that the electropositive H atoms can be aligned to the AlGaN surface. The applied voltage should be larger than the coercive voltage of 60 V for P(VDF-TrFE), so that the P(VDF-TrFE) film can be polarized. The maximum applied drive voltage on the source/drain pads was ± 120 V. Ferroelectric hysteresis loops of the P(VDF-TrFE) film on AlGaN/GaN MOS-HEMTs were measured and shown in Fig. 3(b). As shown in Fig. 3(b), the measured remnant polarization *P*_*r*_ of P(VDF-TrFE) on the AlGaN/GaN MOS-HEMTs is around 2.5 μC/cm^2^, which is smaller than the 4.8 μC/cm^2^ obtained from the P(VDF-TrFE) test structure. This could be due to the asymmetry of the electrodes used (one is Au and the other is Ti/Al pad), as compared to those used in the P(VDF-TrFE) test structure^36^.

**Figure 4:**
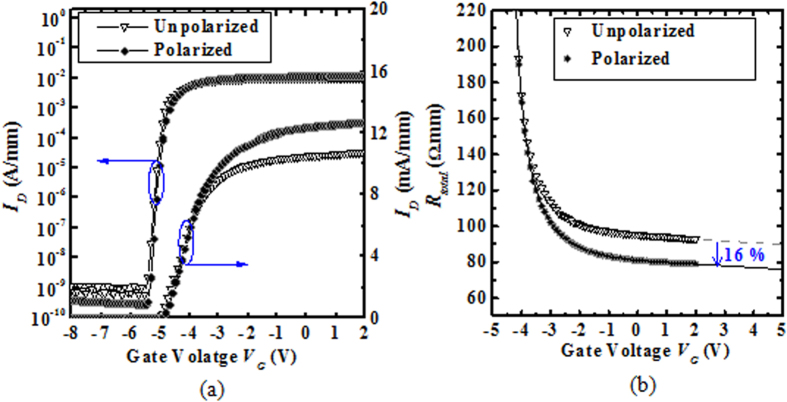
(**a**) *I*_*D*_ – *V*_*G*_ (left: semi-log scale, and right: linear scale) transfer characteristics at *V*_*D*_ = 1 V of AlGaN/GaN MOS-HEMTs with unpolarized and (±120 V) polarized P(VDF-TrFE) gating. *V*_*th*_ is −4.8 V for both devices. Both polarized and unpolarized results came from the same device. (b)Total resistance *R*_*Total*_ (*V*_*D*_ = 1 V) as a function of gate voltage *V*_*G*_ for AlGaN/GaN MOS-HEMTs with unpolarized and (±120 V) polarized P(VDF-TrFE) gating.

Figure 4(a) shows the *I*_*D*_ – *V*_*G*_ transfer characteristics of the AlGaN/GaN MOS-HEMTs with unpolarized and (±120 V) polarized P(VDF-TrFE) gating. The sub-threshold swing *S* did not degrade after the polarization of the P(VDF-TrFE) gating, which is 80 mV/decade in each case. There is also no change in the threshold voltage *V*_*th*_, which is around −4.8 V for both cases. This is expected since the polarization of the P(VDF-TrFE) gating only modulates the 2-DEG density in the access regions. The total resistance *R*_*Total*_ is defined here as the resistance measured between the source and drain pads of the device using a small drain voltage (say *V*_*D*_ = 1 V) and under a large applied gate voltage *V*_*G*_. The value of the source/drain series resistance *R*_*S/D*_is defined as *R*_*S/D*_ = *R*_*Total*_ – *R*_*Channel*_, where *R*_*Channel*_ is the resistance of the channel under the gate. For a very large gate-overdrive *V*_*G*_ - *V*_th_ (*V*_*G*_*-V*_*th*_ ≫ *V*_*D*_) with a small fixed *V*_*D*_, *R*_*Channel*_ becomes very small compared to *R*_*S/D*_, and *R*_*S/D*_ can be estimated from the *R*_*Total*_ versus *V*_*G*_ plot. As shown in Fig. 4(b), the source/drain series resistance *R*_*S/D*_is reduced from 90.8 Ω.mm to 76.7 Ω.mm after polarizing P(VDF-TrFE) gating, or by 16% for the AlGaN/GaN MOS-HEMTs with the (±120 V) polarized P(VDF-TrFE) gating, as compared to that of the AlGaN/GaN MOS-HEMTs with the unpolarized P(VDF-TrFE) gating.

*R*_*S/D*_, which is attributed by the contact resistance and the resistance of the source/drain access region, can be estimated using the equation below:


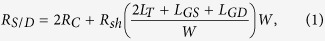


where *R*_*C*_ is the contact resistance, *R*_*sh*_ is the sheet resistance of source/drain access region, *L*_*T*_ is the transfer length from source and drain contact pads, and *W* is the device width (70 μm). From the fabricated transmission line method (TLM) test structure without the P(VDF-TrFE) film, a contact resistance *R*_*C*_ of 10.8 Ω.mm and a transfer length *L*_*T*_ of 29 μm were obtained^37^. Here, it is assumed that the contact resistance *R*_*C*_was not affected by the P(VDF-TrFE) film, since the P(VDF-TrFE) gating is used to modulate only the resistance of the source/drain access region. With a known source/drain series resistance *R*_*S/D*_, a contact resistance *R*_*C*_, and a transfer length *L*_*T*_, the sheet resistance *R*_*sh*_ of source/drain access region for the AlGaN/GaN MOS-HEMTs with unpolarized and (±120 V) polarized P(VDF-TrFE) gating can be estimated to be 887 Ω/□ and 706 Ω/□, respectively, using the Equation (1). The bulk resistivity can be estimated by using the following Equation:


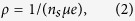


where *ρ* is the bulk resistivity, *n*_*s*_ is the carrier density, *μ* is the carrier mobility, and *e* is the magnitude of electronic charge^38^. The bulk resistivity *ρ*_0_of the AlGaN/GaN heterostructure without the P(VDF-TrFE) gating is estimated to be 488 Ω.cm using Equation (2), with a given electron mobility *μ*_*n*_ of 1600 cm^2^*/*V·s and the^2^-DEG density *n*_s_ of 8 × 10^12^ cm^−2^. Since the sheet resistance *R*_*sh*_ is proportional to the resistivity *ρ*, resistivity *ρ*_1_of AlGaN/GaN heterostructure with the polarized P(VDF-TrFE) gating can be estimated using the relationship below:


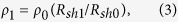


where *R*_*sh*0_ is the sheet resistance of AlGaN/GaN heterostructure without the P(VDF-TrFE) gating and *R*_*sh*1_ is the sheet resistance of AlGaN/GaN heterostructure with the polarized P(VDF-TrFE) gating. The bulk resistivity *ρ*_1_ is calculated to be 388 Ω.cm using Equation (3). Using the simulated 2-DEG density *n*_s_ of 13.6 × 10^12^ cm^−2^ and the calculated resistivity of 388 Ω.cm for the AlGaN/GaN MOS-HEMTs with the polarized P(VDF-TrFE) gating, its electron mobility can be estimated to be 1184 cm^2^/V·s. This is smaller than that of the device with the non-polarized P(VDF-TrFE) gating. The polarized P(VDF-TrFE) gating in the source/drain access region not only increases the 2-DEG density, but also decreases the electron mobility, which could be attributed to an increase of electron-eletron columb scateering within the 2-DEG channel. Depending on the roughness of the AlGaN/GaN interface, the decrease of electron mobility at very high 2-DEG density (more than 10^13^ cm^−2^) could be due to an increase in the interface roughness scattering, since the average distance of the 2-DEG to the AlGaN/GaN interface becomes smaller for a very high 2-DEG density^39^.

Source/drain series resistance reduction for AlGaN/GaN MOS-HEMTs using a polarized P(VDF-TrFE) ferroelectric polymer gating in the access regions is reported in this work for the first time. A crystalline β-phase P(VDF-TrFE) film was formed after baking at 135 °C for 20 hours, and large remnant polarization and high coercive voltage for P(VDF-TrFE) were obtained. For the AlGaN/GaN MOS-HEMTs with a positively polarized P(VDF-TrFE) polymer gating, the conduction band of AlGaN layer is shifted downward due to the polarization of the P(VDF-TrFE) film, resulting in the lowering of the conduction band at AlGaN/GaN interface, thereby increasing the 2-DEG density in the triangular quantum well. The effect of the polarized P(VDF-TrFE) gating in the source/drain access region not only increases the 2-DEG density, but also decreases the electron mobility possibly due to increased AlGaN/GaN interfacial scattering or electron-electron coulomb scattering. Overall, however, the total series resistance is reduced for the AlGaN/GaN MOS-HEMTs with the positively polarized P(VDF-TrFE) gating. To conclude, series resistance reduction can be achieved in AlGaN/GaN MOS-HEMT using a polarized P(VDF-TrFE) gating.

## Methods

### Preparation and characterization of P(VDF-TrFE) film

The P(VDF-TrFE) solutions (3 wt%) were prepared by dissolving P(VDF-TrFE) (70/30% mol) powder in mixed solvent of dimethylformamide (DMF) and Acetone(50: 50 in volume). The solutions were stirring at 1000 rpm for 2 hours at 50 °C on the hot plate. The thin films were deposited by spin coating the solutions at 1000 rpm for 20 s. The thin films were dried at 70 °C for one hour then transferred to oven and bake at 135 °C for 20 hours to form β-phase. AFM and XRD were employed to characterize the surface morphology and crystal structure of P(VDF-TrFE) films.

### Fabrication and characterization of AlGaN/GaN MOS-HEMTs

The AlGaN/GaN structure was grown by metal-organic chemical vapor phase deposition (MOCVD) on a 2-inch sapphire substrate. The epitaxial layers consist of a 25 nm undoped Al_0.25_Ga_0.75_N barrier layer formed on a 2.7 μm undoped GaN layer, which was grown on a 300 nm Fe-doped GaN buffer layer. The electron Hall mobility *μ*_*n*_ and the 2-DEG density *n*_s_ were measured to be 1600 cm^2^/V·s and 8 × 10^12^ cm^−2^, respectively. The fabrication process includs mesa isolattion by Cl_2_ (10 sccm)/BCl_3_ (20 sccm) reactive ion etching (RIE), gate dielectric deposition (15 nm Al_2_O_3_) by atomic layer deposition (ALD), gate metal (100 nm TaN) by magnetron sputtering system, gate electrode definition using standard photolithography, the source/drain contacts [Ti (20 nm)/Al (120 nm)/Ti (10 nm)/Pt (100 nm)] deposition by E-beam, alloying process (650 °C, 30 s) in N_2_ ambient. P(VDF-TrFE) with a 75/25 molar ratio was spin-coated on the devices and baked at 135 °C for 20 hours. An 80 nm-thick gold (Au) film was then deposited by sputtering and patterned as an electrode for the P(VDF-TrFE). Gold is a chemically inert metal, and can avoid the reaction with the P(VDF-TrFE) film to form an non-ferroelectric layer near the interface between gold and the P(VDF-TrFE)^40^. The device in this work has a gate length *L*_*G*_ of 2 μm, a gate-to-source distance *L*_*GS*_ of 5 μm and a gate-to-drain distance *L*_*GD*_ of 15 μm. The ferroelectric hysteretic measurement on the test structure and devices were performed by using Radiant Technology Precision LC.

## Additional Information

**How to cite this article**: Liu, X. *et al*. AlGaN/GaN Metal-Oxide-Semiconductor High-Electron-Mobility Transistor with Polarized P(VDF-TrFE) Ferroelectric Polymer Gating. *Sci. Rep*. **5**, 14092; doi: 10.1038/srep14092 (2015).

## Figures and Tables

**Table 1:** Comparison of P(VDF-TrFE) with other ferroelectric materials.

Ferroelectric material	P(VDF-TrFE)	PbTiO_3_	SrBi_2_Nb_2_O_9_	BiFeO_3_
**Remnant Polarization** ***P***_***r***_ **(mC/cm**^2^)	4.8	53	11.46	95
**Coercive Field** ***E***_***C***_ **(MV/cm)**	1.2	0.75	0.034	0.012
**Deposition Method**	Spin coating	MBE^1^	PLD^2^	LDMOCVD^3^
**Deposition Temperature (°C)**	25	600 ~ 650	400	630
**Reference**	This work	28	29	30

Liu *et al*.

^1^MBE: Molecular Beam Epitaxy.

^2^PLD: Pulsed Laser Deposition.

^3^LDMOCVD: Liquid Delivery Metal Organic Chemical Vapor Deposition.

## References

[b1] Pearton S. J. & Ren F. GaN electronics. Adv. Mater. 12, 1571–1580 (2000).

[b2] Zhang N.-Q. . High breakdown GaNHEMT with overlapping gate structure. IEEE Electron Device Lett. 21, 42–423 (2000).

[b3] Khan M. A., Kuznia J. N., Bhattarai A. R. & Olson D. T. Schottky-barrier photodetector based on mg-doped p-type gan films. Appl. Phys. Lett. 63, 2455–2456 (1993).

[b4] Khan M. A., Bhattarai A., Kuznia J. N. & Olson D. T. High-electron-mobility transistor based on a GaN-Al_x_Ga_1-x_N heterojunction. Appl. Phys. Lett. 63, 1214–1215 (1993).

[b5] Lee D. S. . Impact of GaN channel scaling in InAlN/GaN HEMTs. in IEDM Tech. Dig. 10.1109/IEDM.2011.6131583.

[b6] Zhou Q. . Schottky source/Drain Al2O3/InAlN/GaN MIS-HEMT with Steep Sub threshold swing and high on/off current ratio. 10.1109/IEDM.2011.6131664.

[b7] Yuan L., Chen H. & Chen K. J. Normally-off AlGaN/GaN metal-2DEG tunnel-junction field-effect transistors. IEEE Electron Device Lett. 32, 303–305 (2011).

[b8] Tang Y. . High-performance monolithically-integrated E/D mode InAlN/AlN/GaN HEMTs for mixed-signal applications. in IEDM Tech. Dig. 10.1109/IEDM.2010.5703451.

[b9] Chung J. W., Kim T.-W. & Palacios T. Advanced gate technologies for state-of-the-art f_(T)_ in AlGaN/GaN HEMTs. *in IEDM Tech*. Dig. 10.1109/IEDM.2010.5703449.

[b10] Shinohara K. . 220GHz f_T_ and 400GHz f_max_ in 40-nm GaN DH-HEMTs with re-grown ohmic. *in IEDM Tech*. Dig. 10.1109/IEDM.2010.5703448.

[b11] Marcon D. . A comprehensive reliability investigation of the voltage-, temperature- and device geometry-dependence of the gate degradation on state-of-the-art GaN-on-Si HEMTs. in IEDM Tech. Dig. 10.1109/IEDM.2010.5703398.

[b12] Liu X. . Diamond-like carbon (DLC) liner with highly compressive stress formed on AlGaN/GaN MOS-HEMTs with *in situ* silane surface passivation for performance enhancement. in IEDM Tech. Dig. 10.1109/IEDM.2010.5703340.

[b13] Ota K. . A normally-off GaN FET with high threshold voltage uniformity using a novel piezo neutralization technique. in IEDM Tech. Dig. 10.1109/IEDM.2009.5424398.

[b14] Chu R. . 1200-V normally off GaN-on-Si field-effect transistors with low dynamic on-resistance. IEEE Electron Device Lett. 32, 632–634(2011).

[b15] Lu B. & Palacios T. High breakdown (>1500 V) AlGaN/GaN HEMTs by substrate-transfer technology. IEEE Electron Device Lett. 31, 951–953 (2010).

[b16] Dora Y. . High breakdown voltage achieved on AlGaN/GaN HEMTs with integrated slant field plates. IEEE Electron Device Lett. 27, 713–715 (2006).

[b17] Tipirneni N. . The 1.6-kV AlGaN/GaN HFETs. IEEE Electron Device Lett. 27, 716–718 (2006).

[b18] Treidel E. B. . AlGaN/GaN/GaN:C back-barrier HEMTs with breakdown voltage of over 1 kV and low R_ON_ × A. IEEE Trans. Electron Devices. 57, 3050–2058 (2010).

[b19] Treidel E. B. . AlGaN/GaN/AlGaN DH-HEMTs breakdown voltage enhancement using multiple grating field plates (MGFPs). IEEE Trans. Electron Devices. 57, 1208–1216 (2010).

[b20] Saito W., Takada Y., Kuraguchi M., Tsuda K. & Omura I. Recessed-gate structure approach toward normally off high-Voltage AlGaN/GaN HEMT for power electronics applications. IEEE Trans. Electron Devices 53, 356–362 (2006).

[b21] Uemoto Y. . A Normally-off AlGaN/GaN Transistor with R_on_A = 2.6mΩcm^2^ and BV_ds_ = 640V using conductivity modulation. in IEDM Tech. Dig. 10.1109/IEDM.2006.346930.

[b22] Lee H. S. . InAlN/GaN MOSHEMTs with AlGaN back barrier. IEEE Electron Device Lett. 33, 982–984 (2012).

[b23] Ikeda N. . Highpower AlGaN/GaN MIS-HFETs with field-plates on Si substrates. in Proc. 21^st^ Int. Symp. Power Semicond. Devices IC’s, 10.1109/ISPSD.2009.5158049.

[b24] Selvaraj S. L., Watanabe A., Wakejima A. & Egawa T. 1.4-kV breakdown voltage for AlGaN/GaN high-electron-mobility transistors on silicon substrate. IEEE Electron Device Lett. 33, 1375–1377 (2012).

[b25] Uemoto Y., Ueda T., Tanaka T. & Ueda D. Recent advances of high voltage AlGaN/GaN power HFETs. in Proc. Gallium Nitride Mater. Devices IV. 10.1117/12.808817.

[b26] Wang Z.-G. . Modulation of 2DEG in AlGaN/GaN heterostructure by P(VDF-TrFE). Semicond. Sci. Technol. 26, 125010 (2011).

[b27] Stolichnov I., Malin L., Muralt P. & Setter N. Ferroelectric gate for control of transport properties of two-dimensional electron gas at AlGaN/GaN heterostructures. Appl. Phys. Lett. 88, 043512 (2006).

[b28] Zhang C. J. . Growth and structure of MBE-grown PbTiO_3_ epilayers by using RF atomic oxygen source. J. Cryst. Growth 312, 382–385 (2010).

[b29] Yang P., Carroll D. L., Ballato J. & Schwartz R. W. Growth and optical properties of SrBi_2_Nb_2_O_9_ ferroelectric thin films using pulsed laser deposition. J. Appl. Phys. 93, 9226–9230 (2003).

[b30] Yang S. Y. . Capacitance-voltage characteristics of BiFeO_3_/SrTiO_3_/GaN heteroepitaxial structures. Appl. Phys. Lett. 91, 022909 (2007).

[b31] Kananura M. . Enhancement-mode GaN MIS-HEMTs with n-GaN/i-AlN/n-GaN triple cap layer and high-gate dielectrics. IEEE Electron Device Lett. 31, 189–191 (2010).

[b32] Ohki T. . An over 100W AlGaN/GaN enhancement mode HEMT power amplifier with piezoelectric induced cap structure. Phys. Status Solidi C 6, 1365–1368 (2009).

[b33] Zheng Y. . Graphene field-effect transistors with ferroelectric gating. Phys. Rev. Lett. 105, 166602 (2010). 21230990 10.1103/PhysRevLett.105.166602

[b34] Zheng Y. . Gate-controlled nonvolatile graphene-ferroelectric memory. Appl. Phys. Lett. 94, 163505 (2009).

[b35] Smorchkova I. P. . Polarization-induced charge and electron mobility in AlGaN/GaN heterostructures grown by plasma-assisted molecular-beam epitaxy. J. Appl. Phys. 86, 4520–4526 (2009).

[b36] Xia F. & Zhang Q. M. Schottky emission at the metal polymer interface and its effecton the polarization switching of ferroelectric poly (vinylidenefluoride-trifluoroethylene) copolymer thin films. Appl. Phys. Lett. 85, 1719–1721 (2004).

[b37] Liu X. . AlGaN/GaN metal–oxide–semiconductor high-electron-mobility transistors with a high breakdown voltage of 1400V and a complementary metal–oxide–semiconductor compatible gold-free process. Jpn. J. Appl. Phys. 52, 04CF06 (2013).

[b38] Schroder D. K. Semiconductor Material and Device Characterization. 1–3 (2^nd^ ed. Wiley, New York, 2008).

[b39] Ambacher O. . Two-dimensional electron gases induced by spontaneous and piezoelectric polarization charges in N-and Ga-face AlGaN/GaN heterostructures. J. Appl. Phys. 85, 3222–3233 (1999).

[b40] Naber R. C. G., Asadi K., Blom P. W. M., Leeuw D. M. D. & Boer B. D. Organic nonvolatile memory devices based on ferroelectricity. Adv. Mater. 22, 933–945 (2010). 20217816 10.1002/adma.200900759

